# Cocaine Hurts Your Kidneys Too: A Rare Case of Acute Interstitial Nephritis Caused by Cocaine Abuse

**DOI:** 10.7759/cureus.19236

**Published:** 2021-11-03

**Authors:** Tahmina Jahir, S.M. Sadaf Hossain, Ruby Risal, Marie Schmidt, Danilo Enriquez, Mobasera Bagum

**Affiliations:** 1 Pulmonary and Critical Care Medicine, Interfaith Medical Center, Brooklyn, USA; 2 Internal Medicine, Jamaica Hospital Medical Center, Richmond Hill, USA; 3 Pulmonary Medicine, Interfaith Medical Center, Brooklyn, USA; 4 Pulmonology, Interfaith Medical Center, Brooklyn, USA; 5 Internal Medicine, Interfaith Medical Center, Brooklyn, USA

**Keywords:** acute interstitial nephritis, non-traumatic rhabdomyolysis, acute renal failure and hemodialysis in icu, hemodialysis, cocaine

## Abstract

Cocaine is a powerfully addictive recreational drug that is extracted from the leaves of the *Erythroxylon coca* plant native to Central and South America. It is a widely abused substance, despite being banned since the early 19th century due to fatalities. Cocaine may result in acute kidney injury (AKI) by different mechanisms, but acute interstitial nephritis (AIN) is scarcely recognized as the cause of acute kidney injury (AKI). Here, we present a case of AKI from both AIN and acute tubular necrosis (ATN) following cocaine insufflation. The purpose of this article is to review the rare but significant association of AIN associated with cocaine use. The nature of the treatment of cocaine-related kidney disease may differ from other causes of acute kidney insult. Prompt recognition of the underlying cause of renal dysfunction is vital for this group of patients to prevent the rapid deterioration of renal function.

## Introduction

Cocaine-related kidney damage has been reported previously. This illicit drug is a potent activator of the sympathetic nervous system leading to intense systemic vasoconstriction, endothelial dysfunction, platelet activation, oxidative stress, and decrease in prostaglandins E2 and prostacyclin which may lead to multiorgan failure [1,2]. Cocaine toxicity may result in acute kidney injury (AKI) via tubular necrosis due to vasoconstriction or myoglobin-induced renal injury secondary to rhabdomyolysis. However, acute interstitial nephritis (AIN) is scarcely recognized as a potential cause of acute kidney injury (AKI) in cocaine users [1,2]. Here, we present a case of a 60-year-old African American man who developed AKI from both AIN and acute tubular necrosis (ATN) after cocaine insufflation via the nose, and we review the literature on the association between cocaine use and the development of AIN. This study aimed to recognize the potential complications of cocaine-related toxicity in the kidney and a brief discussion on the rare, reportable, and significantly important association between cocaine use and the development of AIN.

## Case presentation

A 60-year-old African American male with a past medical history of cardiac arrest due to cocaine overdose 20 years ago, hypertension, asthma, tobacco use disorder, and cocaine use disorder presented to ED with a chief complaint of malaise and generalized weakness for one day. It was associated with loss of appetite, abdominal distension without nausea or vomiting for the same duration. He started to binge on alcohol and cocaine for five to seven days before presentation. After ED arrival, the patient was awake but in mild distress secondary to abdominal discomfort. Vital signs were significant for desaturation to 92% on room air, tachycardia of 108 beats/min, and borderline low blood pressure of 108/67 mmHg. Physical examination was remarkable for dry oral mucous membrane, dry and scaly skin, and moderate tenderness on palpation of the left lower quadrant of the abdomen. 

Labs drawn in the emergency department showed significant neutrophilic leukocytosis with left shift white blood cell (WBC) 26.7 x 10^3^/uL, hemoglobin of 17.2 gm/dL, hematocrit of 52%, and platelet of 360 x 10^3^/uL. Chemistry showed mild hypernatremia of 150 mg/dL, potassium of 4 mEq/L (4 mmol/L), chloride of 102 mEq/L (93 mmol/L), low bicarbonate of 15 mEq/L with significantly elevated blood urea nitrogen (BUN) of 54.3 mg/dL and creatinine (Cr) of 6.38 mg/dL. This patient's last BUN and Cr levels were normal one year ago. Labs also reported a significantly elevated anion gap of 31, arterial blood gas showed pH of 7.20, PaCO_2_ of 39.3 mmHg PaO_2_ of 139 mmHg on 2 L nasal cannula, calculated bicarbonate of 17.0 mEq/L, with elevated lactic acid 4 mmol/L, serum osmolarity of 321 mosmol/L, no osmolar gap, creatinine phosphokinase of >36,000 u/L with cola color urine suggestive of severe rhabdomyolysis. Chemistry also showed significantly deranged liver function tests (LFTs), aspartate aminotransferase (AST) 2298 u/L, alanine aminotransferase (ALT) 598 u/L, AST>ALT, total bilirubin 2.3 mg/dL, alkaline phosphatase (ALP) 142 U/L, lactate dehydrogenase (LDH) >4300 U/L with significantly elevated D-dimer of >18,000 ng/mL (Table 1). The patient's urine toxicology screen panel came positive for cocaine, other toxicology screening was unremarkable including Tylenol, salicylate, cannabinoid, opioid, and alcohol (Table 2). Urine analysis showed increased specific gravity of 1.030, large blood 4+, >100 red blood cells (RBC), small leukocyte esterase positive, >20 white blood cells/high-power field, positive for nitrate and moderate bacteria, suggestive of complicated urinary tract infection (UTI). Urine analysis also showed moderate amorphous sediments with muddy brown cast and WBC cast, urine eosinophils suggestive of acute tubular necrosis (ATN), and AIN. Calculated fractional excretion of sodium (FENa) on admission was 3.3%, suggestive of Intrinsic renal abnormality. An abdominal CT scan showed patchy hypoenhancement of the kidneys, suggestive of sequelae of hypoperfusion or pyelonephritis. The patient was placed on a nasogastric tube due to a distended abdomen and 200 mL of coffee ground emesis came out which was positive for blood. 

**Table 1:** Summary of the laboratory testing done on the day of admission. BUN: blood urea nitrogen

Labs	Value	Reference
Serum sodium	150 mmol/L	136-145 mmol/L
Serum magnesium	4.5 mg/dL	1.6-2.6 mg/dL
Serum phosphorus	8.5 mg/dL	2.3-4.7 mg/dL
Serum calcium	6.5 mg/dL	8.4-10.2 mg/dL
Serum uric acid	20.9 md/dL	3.8-8.4 mg/dL
Serum creatinine	6.38 mg/dL	0.72-1.25 mg/dL
Serum BUN	51.0 mg/dL	8.4-25.7 mg/dL
Serum pH	7.1 unit	7.350-7.450 unit
Serum anion gap	31	8-16
Serum bicarbonate	17.0 mmol/L	18-24 mmol/L
Serum lactate	4 mmol/L	0.5-1.9 mmol/L
Lactate dehydrogenase	4300 U/L	125-220 U/L
Creatinine kinase	>360,000 U/L	30-200 U/L
Serum troponin	824.8 ng/L	0.0-35.0 ng/L
B-type natriuretic peptide	188.69 pg/mL	10-100 pg/mL
Serum D-dimer	6380 ng/mL	45-500 ng/mL
Alanine aminotransferase (ALT)	443 U/L	10-55 U/L
Aspartate aminotransferase (AST)	1923 U/L	5-34 U/L
White blood count	26.7 uL	4.5-11.0 uL
Neutrophils	89.7%	40-70%
Serum iron	15 ug/dL	38-169 ug/dL
Total iron-binding capacity	129 ug/dL	250-450 ug/dL

**Table 2:** Creatinine trend throughout hospitalization. BUN: blood urea nitrogen

	Day 1	Day 2	Day 3	Day 4	Day 5	Day 6	Day 7	Day 8	Day 9	Day 10
BUN (mg/dL)	54.3	76	78.4	85.9	67.5	78.1	6.8	49.1	14.7	17
Creatinine (mg/dL)	6.2	7.47	7.69	8.13	6.62	8.21	0.68	10.25	1.33	1.34

The patient was admitted to the medical ICU for acute renal failure, acute hypercarbic respiratory failure, high anion gap metabolic acidosis with lactic acidosis (HAGMA), and acute liver injury, likely secondary to cocaine toxicity (Table 1). The patient was given 3 L intravenous (IV) isotonic normal saline followed by starting on maintenance IV fluid and bicarbonate drip for severe metabolic acidosis. A foley catheter was placed to closely monitor the intake and output however he was found anuric. He was started on broad-spectrum antibiotic coverage with vancomycin and meropenem for complicated UTI and IV pantoprazole drip was given for acute upper GI bleeding. 

**Figure 1:**
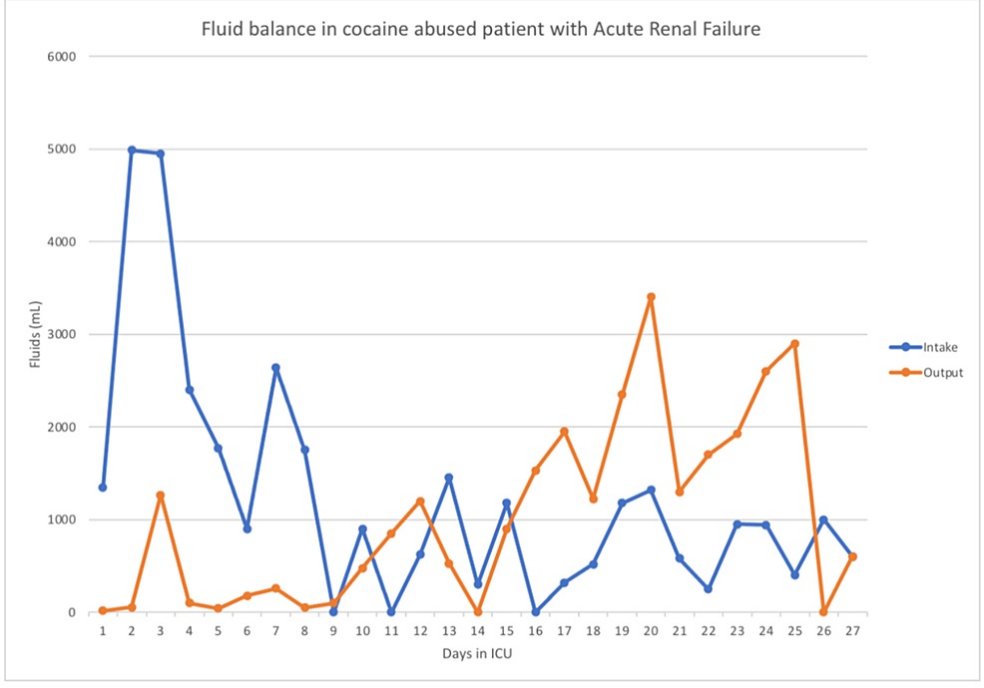
Intake and output monitoring graph. The output curve represents urine output, stool output as well as suction fluid from the patient's nasogastric tube. On day two, the patient had a nasogastric aspirate and stool output amounting to about 1.5 L. The patient began producing urine around day nine. The patient continued to receive IV fluids; however, it is not reflected in the curve due to lack of documentation.

Despite resuscitation with IV fluid on the first day of ICU admission, liver enzymes, creatinine kinase, and BUN/Cr kept trending up. Repeat labs showed creatine phosphokinase (CPK) of 158671 U/L, AST of 2298 U/L, ALT of 597 U/L, BUN of 85.9 mg/dL, Cr of 8.21 mg/dL, bicarbonate further <10 mmol/L and potassium of 6.5 mmol/L, phosphorus of 6.5 mmol/l, and decreased in the calcium of 6.6 mg/dL. An emergency dialysis catheter was inserted and hemodialysis was started on the second day of ICU admission. On day ninth, he started making urine. During the hospital course, GI bleeding stopped, abdominal pain and distension resolved. After multiple sessions of hemodialysis patients, creatine kinase (CK) started dropping with improvement in BUN and creatinine function slowly along with improvement in urine output (Table 3, Figure 1). Detailed intake and output can be seen in Figure 1. He started tolerating the PO diet as well. After the 20th day, his BUN and Cr went down to 08/1.23, hemodialysis was discontinued, and he was discharged home. 

**Table 3:** Summary of the urine toxicology screening done on the day of admission.

Urine drug screen	Result	Reference
Opiate	Negative	Absent
Methadone	Negative	Absent
Propoxyphene	Negative	Absent
Barbiturates	Negative	Absent
Phencyclidine	Negative	Absent
Amphetamines	Negative	Absent
Benzodiazepines	Negative	Absent
Cocaine	Positive	Absent
Cannabinoids	Negative	Absent
Ethyl alcohol	Negative	Absent

## Discussion

Cocaine is abused worldwide which is an activator of the sympathetic nervous system leading to intense vasoconstriction, endothelial dysfunction, oxidative stress, platelet activation, and decrease in prostaglandins E2 and prostacyclin which create widespread systemic adverse effects [1,2]. It inhibits catecholamine reuptake and moderate release and reuptake-blockade of serotonin and dopamine which may lead to irreversible organ damage by overstimulation of the adrenergic system. 

One of the most common adverse effects of cocaine abuse is acute kidney injury (AKI) which occurs by different mechanisms like rhabdomyolysis, vasculitis, infarction, thrombotic microangiopathy, and malignant hypertension. One of the rare and seldom reported causes of cocaine-induced AKI is AIN. This study aimed to review the causes of cocaine-induced AKI and raise awareness among physicians about the rare but reportable association between cocaine toxicity and the development of AIN. 

Cocaine-induced rhabdomyolysis is defined as both traumatic (from cocaine-induced seizure or hyperpyrexia) and non-traumatic injury to skeletal muscle (from vasoconstriction) which leads to muscle ischemia and necrosis [3,4]. This results in the release of myoglobin which has the potential to cause AKI by renal vasoconstriction with resultant ischemia, free radical generation, and direct cytotoxicity on proximal tubular tubules via heme pigment leading to muddy brown casts formation.

Acute interstitial nephritis (AIN) affects renal function by an inflammatory infiltrate in the kidney interstitium. The most common etiology of AIN is drug-induced, which includes 60-70% of cases. AIN is characterized by interstitial inflammation, tubulitis, edema, and interstitial fibrosis. A patient with AIN presents with elevated serum creatinine and a urinalysis that shows white cells, white cell casts, and, in some cases, eosinophiluria [5]. Although kidney biopsy is the key for definitive diagnosis, It is often considered unnecessary to make a definitive diagnosis when clearly documented onset of kidney failure occurs after initiation of an offending drug and improves immediately after discontinuation of that particular drug-like in our case. It is very important to remember while diagnosing AIN, the classic triad of fever, rash, and eosinophilia is only present in one-third of patients and the absence of these findings should not be used to exclude the diagnosis. The mainstay of therapy for drug-induced AIN is the discontinuation of the offending agent. Although the benefits of corticosteroid therapy remain unproven so far. The drug-induced AIN has a good prognosis, and full recovery of kidney function is usually observed like in our case [5,6]. That's why early recognition is crucial to treat reversible causes, otherwise, patients may ultimately develop chronic kidney failure and require permanent hemodialysis [6,7]. Recently, a few case reports have been published suggesting a link between AIN and cocaine exposure in patients presenting with AKI and cocaine abuse. In our case, our patient had developed cocaine-induced severe AKI from both rhabdomyolysis and AIN, and early recognition and rapid initiation of hemodialysis has fully reversed his kidney function and avoided permanent kidney damage [7].

## Conclusions

When considering a diagnosis of AIN, a thorough drug history is prudent. The cause of cocaine-induced AKI is multifactorial; however, AIN related to cocaine is rare but noteworthy in raising awareness among physicians. This is because its early recognition will help avoid significant deterioration of the patient's renal function. Population-based studies are needed to further assess the magnitude of this pathological association. It will not only broaden the scope of our knowledge on this issue but will also help shape guidelines to standardize the care of such patients.
